# The Role of Gamma Knife Surgery in the Treatment of Rare Sellar Neoplasms: A Report of Nine Cases

**DOI:** 10.3390/cancers17091564

**Published:** 2025-05-03

**Authors:** Michele Longhi, Riccardo Lavezzo, Valeria Barresi, Giorgia Bulgarelli, Anna D’Amico, Antonella Lombardo, Emanuele Zivelonghi, Paolo Maria Polloniato, Giuseppe Kenneth Ricciardi, Francesco Sala, Angelo Musumeci, Giampietro Pinna, Antonio Nicolato

**Affiliations:** 1 Departmental Unit of Stereotaxis, Department of Neurosciences, Hospital Trust of Verona, 37126 Verona, Italy; giorgia.bulgarelli@aovr.veneto.it (G.B.); anna.damico@aovr.veneto.it (A.D.); antonella.lombardo@aovr.veneto.it (A.L.); antonio.nicolato@aovr.veneto.it (A.N.); 2 Unit of Neurosurgery B, Department of Neurosciences, Hospital Trust of Verona, 37126 Verona, Italy; 3 Vertebral Surgery Service, San Lorenzo Hospital-AULSS Berica, 36078 Valdagno, Italy; dott.riccardolavezzo@gmail.com; 4 Unit of Neuropathology, Fondazione IRCSS Carlo Besta, 20021 Milan, Italy; 5 Unit of Medical Physics, Department of Diagnostics and Public Health, Hospital Trust of Verona, 37126 Verona, Italy; emanuele.zivelonghi@aovr.veneto.it (E.Z.); paolomaria.polloniato@aovr.veneto.it (P.M.P.); 6 Unit of Neuroradiology, Department of Diagnostics and Public Health, Hospital Trust of Verona, 37126 Verona, Italy; giuseppe.ricciardi@aovr.veneto.it; 7 Unit of Neurosurgery A, Department of Neurosciences, Hospital Trust of Verona, 37126 Verona, Italy; angelo.musumeci@aovr.veneto.it (A.M.); giampietro.pinna@aovr.veneto.it (G.P.)

**Keywords:** gamma knife, radiosurgery, rare brain tumors

## Abstract

**Introduction:** The group of so-called “sellar-region masses” consists of a heterogeneous group of neoplasms and tumor-mimicking lesions, whose differential diagnosis may be challenging due to the overlapping of clinical and radiological features, which can be found both in “common” and “uncommon” lesions. The choice of a correct treatment strategy is still arduous and requires histological analysis. Gamma Knife Radiosurgery (GKRS) has already been reported as a safe and effective treatment in these cases. The objective of this study is to evaluate single-center pre-operative data, post-operative outcomes, and long-term follow-up in patients treated with GKRS for unusual sellar tumors. **Methods:** We retrospectively identified and analyzed nine patients treated with GKRS from 2004 to 2015, according to a standard protocol. Lesions consist of hypothalamic hamartoma (HH), Rathke’s cleft cist (RCC), Langerhans cell histiocytosis (LCH), spindle cell oncocytoma (SCO), choroid plexus papilloma (CPP), and ossifying fibroma (OF). The diagnosis was histologically confirmed in six patients that underwent surgery, while in three patients, diagnosis was based on characteristic clinical and radiological findings (two HH and one RCC). Pre-operative and post-operative data were retrieved from medical archives, and long-term follow-up was obtained through clinical and neuroradiological periodic examination. **Results:** In our series, all the “rare” sellar lesions treated, had a successful radiographic and clinical response in a medium-long follow-up period. **Conclusions:** The long-term follow-up results suggest that GKRS is a safe and effective treatment in rare sellar lesions, with very low toxicity. To the best of our knowledge, this report represents the largest series of unusual sellar lesions treated with GKRS in a single high-volume center, suggesting that GKRS might be an effective non-invasive adjuvant treatment option. Further studies and a larger number of patients are needed to confirm if residuals of these rare sellar lesions might regress on their own without treatment or if other non-invasive treatments could be as effective as GKRS.

## Simple Summary

This study evaluated the long-term outcomes of Gamma Knife Radiosurgery (GKRS) in treating rare sellar-region lesions at a single high-volume center. Nine patients treated between 2004 and 2015 were retrospectively analyzed. The lesions included hypothalamic hamartoma, Rathke’s cleft cyst, Langerhans cell histiocytosis, spindle cell oncocytoma, choroid plexus papilloma, and ossifying fibroma. Diagnosis was histologically confirmed in six patients, while three were diagnosed based on clinical and radiological features. All cases showed favorable clinical and radiological responses after GKRS, with very low toxicity. These findings suggest that GKRS is a safe and effective non-invasive treatment option even for rare sellar lesions. This represents the largest reported series of such cases treated with GKRS. However, further studies with larger patient cohorts are needed to determine if GKRS offers benefits over the natural course or other non-invasive therapies.

## 1. Introduction

Sellar-region lesions represent a large and heterogenous group of masses including both primitive and metastatic tumors, inflammatory diseases, vascular lesions, and developmental anomalies. The most common lesion is pituitary adenoma (PA), while non-adenomatous sellar lesions are infrequent and account for less than 10% of cases [1,2].

Differential diagnosis may be quite arduous because of the overlapping of clinical, hormonal, and radiological presentation; indeed, sometimes there are no particular findings that are characteristic of a specific non-adenomatous non-secreting lesion besides the histological examination [3]. In this article, we present our experience in the treatment of rare and unusual sellar lesions with Leksell Gamma Knife Radiosurgery from 2004 to 2015. For each case, we provide a brief clinical presentation, the diagnostic pathway, the neurosurgical operation (when performed), the radiosurgical treatment, and the entire follow-up period available from a retrospective analysis of our clinical reports.

The authors thought of proposing Gamma Knife Radiosurgery (GKRS) for these lesions with the aim of dramatically decreasing the risk of post-operative recurrences or progressions—thus avoiding both the need for repeat resections and the risk of further permanent surgery-related complications. The rationale of including these lesions among the indications for GKRS arose from the fact that these lesions could be properly considered benign neoplastic forms. In fact, some authors have suggested there is a close relationship between RCCs and craniopharyngioma since they share a common histological origin from remnants of squamous epithelium from Rathke’s cleft. In addition, the transformation from RCC to craniopharyngioma in the same patient has also been reported [4]. To our knowledge, this is the first series of histologically verified rare and unusual sellar lesions treated with GKRS.

## 2. Material and Methods

More than 16,000 patients underwent Gamma Knife Radiosurgery at AOUI VERONA from 1993 to 2024. Among those, 11 cases of unusual sellar neoplasms were treated with GK and a minimum follow-up of five years was achievable in 9 patients, qualifying them for this study.

The radiosurgical technique has already been described in detail in previous reports.

After the application of the magnetic resonance imaging (MRI)-compatible Leksell Model G stereotactic frame (Elekta Instruments AB, Stockholm, Sweden) to the patients’ heads, the patients underwent stereotactic high-resolution 1.5-Tesla MRI with specific sequences to obtain precise information about the 3D shape of the lesion and the surrounding normal structures. After the acquisition of a survey including axial, coronal, and sagittal images, a post-contrast bolus (Gadobutrol) 2 mm-thick coronal “dynamic” study was performed with the aim of differentiating RCC residuals from pituitary stalks and glands. Post-contrast 1 mm isotropic voxel volumetric scanning, used to define target volume and to determine target coordinates, and axial CISS (Constructive Interference in Steady State) images, to clearly identify the anterior optic pathways, were also acquired. Three-dimensional treatment planning was then developed by using commercially available software, i.e., Leksell Gamma Plan software (version 8.3, Elekta Instruments). The radiosurgery team created a highly conformal dose plan using multiple collimators and performed the dose selection. The mean prescribed dose was 10 Gy normalized to the 50% isodose line. The dose was prescribed to the entire lesion volume as defined using contrast-enhanced imaging. The dosage was selected based on lesion volume, the distance between the lesion and the optic apparatus, and extent of pre-treatment visual deficit. The highest dosage possible in the target was delivered while strictly respecting the known optic nerve tolerance (always kept <12 Gy). For this purpose, detailed evaluation of the volumetric exposure of the anterior optic pathways was performed before treatment. Radiosurgical procedures were performed with Perfexion and the model C 201-source Co^60^ Leksell Gamma Unit (Elekta Instruments AB, Stockholm, Sweden). All Gamma Knife Radiosurgical procedures were performed under local anesthesia according to a standardized protocol; routine monitoring included ECG, pulse oximetry, and patient video monitoring through high-resolution cameras installed inside the Gamma Knife room. The patients’ positioning was the same in all procedures.

Post-radiosurgical course and follow-up. The patients were routinely discharged from hospital within 24 h of treatment. They underwent physical examination, visual field testing, laboratory tests, and serial contrast-enhanced imaging using MR at intervals of 6 months for the first year and annually thereafter. For the evaluation of tumor volume, the mass lesion was measured by using the Gamma Plan software version 8.3.1 and subsequent versions or the OsiriX medical imaging program (version 4.19) developed at the University Hospital of Geneva, Switzerland. Local tumor growth control was defined as the complete disappearance of the lesion, its shrinkage, or stable disease. Long-term follow-up data included clinical and neuroradiological status at time of the last neurosurgical or oncological follow-up.

## 3. Results

In this series of nine patients, with two males and seven females, the mean age was 49 (range 24–78) years. All patients had neoplastic sellar lesions and etiological/radiological diagnoses of hypothalamic hamartoma (HH), Rathke’s cleft cyst (RCC), Langerhans cell histiocytosis (LCH), spindle cell oncocytoma (SCO), choroid plexus papilloma (CPP), and ossifying fibroma (OF). The diagnosis was histologically confirmed in six patients that underwent surgery, while in three patients, diagnosis was based on characteristic radiological findings (two HH and one RCC). The leading symptoms were headache and visual acuity deterioration, followed by fatigue, hormonal disturbance, cranial nerve palsy, epilepsy (particularly gelastic seizures in HH), and diabetes insipidus centralis.

### 3.1. Hypothalamic Hamartoma (n = 3)

With three patients, hypothalamic hamartomas were the most frequent lesions seen in our series.

**Figure 1:**
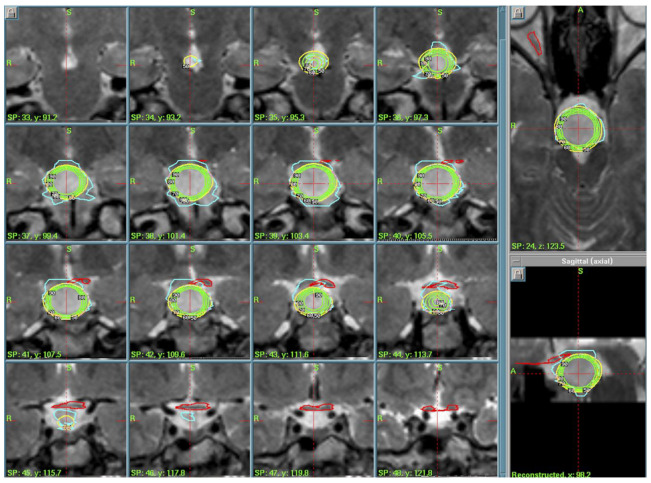
Case 1 treatment and dose-planning in a coronal T2-weighted MRI. In yellow is the 50% isodose line prescribed to the lesion, and in green are the 60% to 90% isodoses. In green and light blue are the contours of the anterior optic pathways and pituitary gland and pituitary stalk.

Case 1: A 24-year-old man presented with a long history of gelastic and adversative seizures. He was diagnosed in the 1990s with slow-growing-rate HH. Due to a loss of seizure control with pharmacological therapy (five drugs), GKRS was proposed after the patient’s surgery refusal. The lesion was relatively large: magnetic resonance images (MRI) consisted of a hamartomatous form of the hypothalamic tuber that rostrally reached the right hemiploom of the third ventricle, forming and determining flow in the right optic hemi-chiasm. Caudally, it pushed into the retro-clival region incorporating the basilar artery. In 2004, a radiosurgical protocol was thus proposed in order to avoid excessive irradiation of optical chiasm and pathways (Peripheral Isodose (PI) of 50%, Prescription Dose (PD) of 11 Gy, Maximal Dose (MD) of 22 Gy, integral dose of 148.7 millijoules (mJ), 12 Gy volume of 7.71 cc). GKRS was suggested due to the patient’s refusal of open surgery and pharmaco-resistant seizures. The lesion was large and located near critical structures (optic chiasm, basilar artery), making radiosurgery a safer alternative. The case was discussed in a multidisciplinary setting. (Figure 1).

The post-operative course was uneventful and the patient was discharged home without new neurological deficit. The patient underwent clinical and radiological follow-up, and the last MRI (August 2020) showed no lesion regrowth, with an absence of pathological enhancement. The patient is now treated with two anti-epileptic drugs (Levetiracetam and Carbamazepine) and shows a good control of residual simple partial seizures. The next FU MRI is scheduled this year.

**Figure 2:**
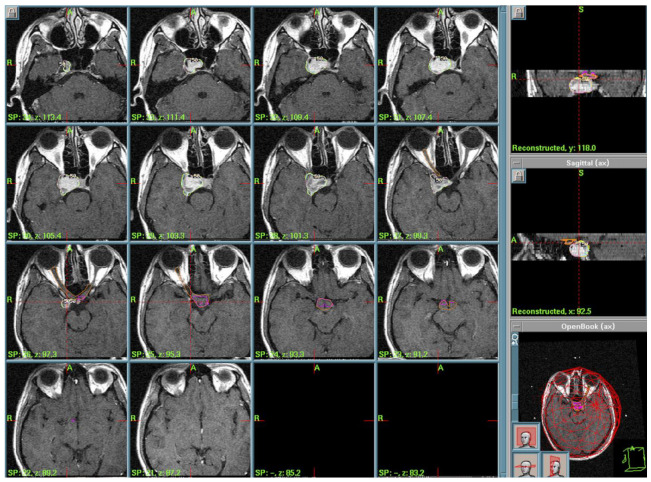
Case 2 treatment planning. Axial view of GammaPlan^TM^ for dose plans. In yellow is the 50% isodose prescribed to the margin of the tumor. In orange and pink is the contouring of the anterior optic pathways to be avoided.

Case 2: A 24-year-old man presented with a 6-month history of diplopia and previously diagnosed hypothalamic hamartoma extended to the right cavernous sinus. The diagnosis was histologically verified after microsurgery (bifrontal craniotomy) at another center. After extemporaneous diagnosis, surgery was interrupted and radiosurgical treatment was proposed. Given the challenging location and risk of complications, adjuvant GKRS was chosen post-multidisciplinary discussion. The treatment’s parameters (2005) were the following: a PI of 50%, PD of 13.5 Gy, MD of 27 Gy, integral dose of 95.2 mJ, and 12 Gy volume of 7.13 cc. (Figure 2). The patient was discharged home the same day of treatment without new neurological deficit. After 5 years of good clinical and radiological follow-up, the patient was no longer contactable.

**Figure 3:**
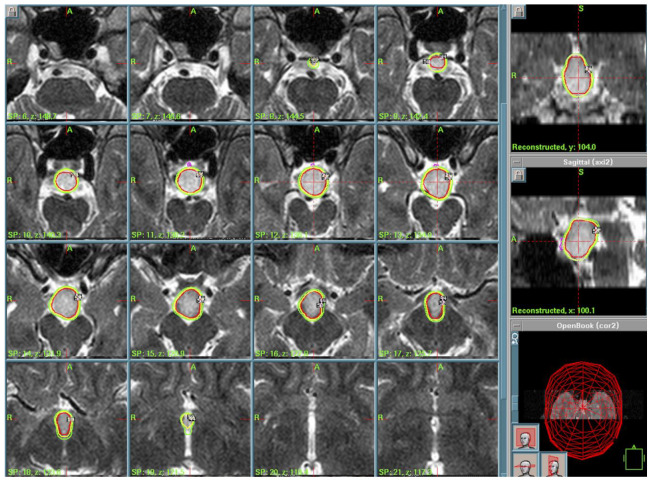
Case 3 treatment planning. Axial T2-weighted MRI view. In red is the contouring of the lesion; in green and yellow is the 50% isodose line, avoiding dose exposure for the brainstem <15 Gy.

Case 3: A 29-year-old man presented with a long history of partial complex and gelastic seizures with mental status changes and aggressiveness in the post-critical phase. MRI revealed a hypothalamic non-enhancing lesion occupying the floor of the third ventricle and mammillary bodies. After adopting a wait-and-see approach, following MRIs showed the slow and progressive growth of the hypothalamic lesion, also involving the sellar region to the optic chiasm. Seizures became pharmaco-resistant, so GKR (PI of 50%, PD of 13 Gy, MD of 26 Gy, integral dose of 51.2 mJ, 12 Gy volume of 4.18 cc) was proposed instead of surgery (due to patient’s refusal and after multidisciplinary evaluation) and performed (2009). (Figure 3) The patient was discharged home after radiosurgery without neurological deficit. Clinical and radiological follow-up showed excellent tumor growth control (August 2019), and the patient did not show any visual field deficit or hormonal impairment. The past neurological evaluation (August 2019) reported good seizure control with two anti-epileptic drugs (Levitiracetam and Valproate).

### 3.2. Pituitary Stalk Langerhans Cell Histiocytosis (LCH)

Case 4: A 47-year-old woman with a clinical history of panhypopituitarism, memory loss, and binocular visual field deterioration was referred to our clinic on February 2007 for adjuvant treatment on residual sellar LCH, one year after neurosurgical operation. MRI showed an expansive lesion involving the floor of the third ventricle that dislocated the optic chiasm and the pituitary stalk. GKRS was used as an adjuvant therapy to avoid a second high-risk surgery procedure. The decision followed interdisciplinary evaluation. Treatment was performed in 2004, with a PI of 50%, PD of 11 Gy, MD of 22 Gy, integral dose of 15.8 mJ/12 mJ, and 12 Gy volume of 1.06 cc/0.65 cc. The treatment was very well tolerated and the patient was discharged home two days after radiosurgery. Follow-up until 2009 was regular and no radiological or clinical deterioration was found. Then, she was lost at follow-up.

### 3.3. Rathke’s Cleft Cist (RCC)

**Figure 4:**
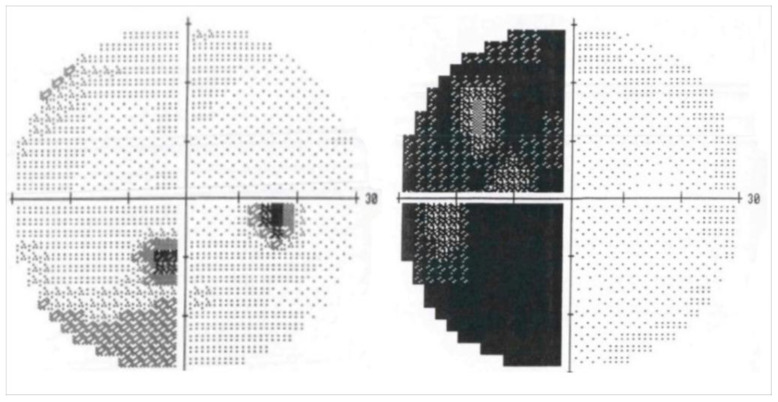
Pre-operative visual field showing incomplete bitemporal hemianopsia.

Case 5: A 45-year-old Italian woman was referred to our clinic on January 2006 with a 6-month history of fatigue, polyuria, polydipsia, hyperprolactinemia, and amenorrhea and was treated with antidiuretic hormone agonist (desmopressin). Contrast enhancement MRI (July 2005) showed a 10 mm, well-defined suprasellar lesion. The lesion did not show apparent enhancement in gadolinium T1-weighted imaging. Over the next two months, the patient began to complain of left temporal hemianopsia, confirmed through visual field examination (Figure 4).

**Figure 5:**
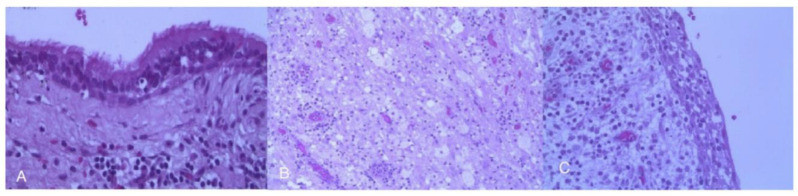
(**A**–**C**) Tissue specimens obtained during the operation were fixed in formalin, embedded in paraffin, sectioned, and stained using hematoxylin and eosin (H-E). Original magnification, ×100 (**A**); original magnification, ×200 (**B**,**C**). A diagnosis of Rahtke’s cleft cyst was confirmed.

New MRI (November 2005) showed an enlargement of the lesion (20 mm) with light dysomogenous enhancement in T1-weighted imaging. Transcranial resection was planned due to the mainly suprasellar location of the lesion believed to be a craniopharyngioma. In January 2006, the patient underwent a bifrontal craniotomy and a partial resection of the lesion was performed via a sub-frontal retro-chiasmatic approach. Tissue specimens obtained during the operation were fixed in formalin, embedded in paraffin, sectioned, and stained using hematoxylin and eosin (H-E). The specimens were then examined with the aid of a light microscope. Histopathological examination revealed that the cyst was lined with ciliated cuboidal and columnar epithelium with no atypia and was filled with mucous-like material. These findings were consistent with typical RCCs (Figure 5A–C).

The were no acute post-op0erative complications. Nevertheless, delayed serum analysis showed the onset of panhypopituitarism, and the patient began treatment with complete hormone substitution.

**Figure 6:**
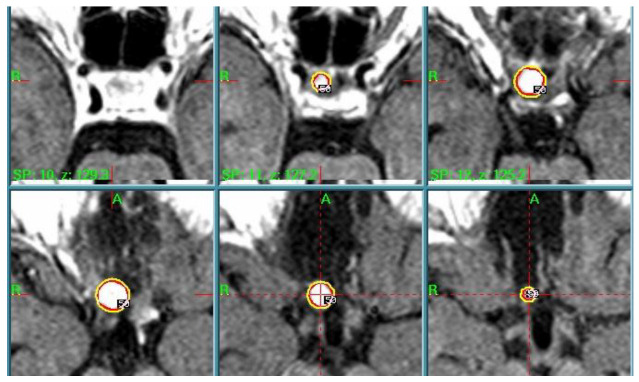
Case 5 treatment planning. The small lesion is identified in T1-weighted MRI (red line) and the 50% isodose line (yellow) is prescribed, showing the high conformation of the dose plan.

A further MR examination performed in December 2006 showed the presence of a 10 mm residual. After multidisciplinary discussion of the clinical case, the radiosurgery team—composed of neurosurgeons, radiation oncologists, and medical physicists—in accordance with the patient decided to treat the RCC remnant with GK. Radiosurgical treatment was performed in March 2007. The dose plan parameters—tailored to a single-session treatment—were a PI of 50%, PD of 12 Gy, MD of 24 Gy, and anterior optic pathway dose always <12 Gy, as described in the literature [5] (Figure 6).

The early post-radiosurgical period was uneventful. Successive follow-up did not show the occurrence of any side effects. Visual field examinations and analysis of serum hormone level confirmed both neuro-ophthalmologic and endocrinologic conditions to be stable. Serial MRI documented a progressive shrinkage of the RCC residual and, at the last available MRI follow-up (July 2014), 88 months after GKRS, neuroradiogical findings showed the complete disappearance of the lesion.

Case 6: A 78-year-old woman presented with a long clinical history of visual deficit and age-related macular degeneration. A visual field campimetry showed a serious deficit on the left eye which could not be related to her macular degeneration. The following MRI revealed a cystic sellar lesion, lifting up the optic chiasm, radiologically suggestive for RCC. Hormonal dosage showed a cortisol deficiency and a prolactin elevation. The patient was elderly and presented comorbidities; GKRS was selected as a non-invasive option due to the lesion’s proximity to the optic chiasm and the patient’s refusal of surgical intervention.

The patient underwent GKRS on November 2015 (PI of 50%, PD of 10 Gy, MD of 20 Gy) and was discharged home the same day of treatment. The last follow-up MRI (October 2023) showed a reduction in the cystic portion of the lesion, and the patient now has a stable visual field deficit without new neurological symptoms.

### 3.4. Ossifying Fibroma (OF) of the Ethmoid Sinus

To our knowledge, there is only on previous report of a juvenile ossifying fibroma treated with adjuvant radiotherapy after surgical excision to improve local growth control [6].

**Figure 7:**
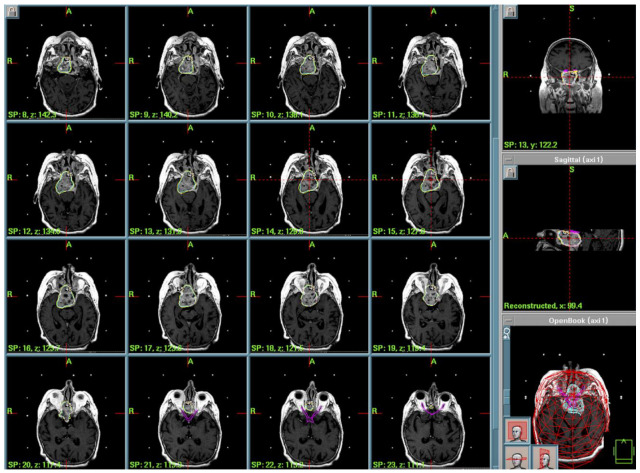
Treatment planning. Volumetric T1-weighted MRI. In yellow is the prescription isodose of the large skull base lesion. In purple is the contouring of the anterior optic pathways.

Case 7: A 70-year-old woman presented with a recent onset of ptosis on the left eye with visual field deficit, diplopia, and frontal headache. MRI showed a voluminous expansive lesion invading sphenoid sinus, with erosion of the clivus and the ethmoid sinus. The patient underwent neurosurgical operation at another center, and histological examination revealed a psammomatoid ossifying fibroma. Post-operative MRI showed residual lesion surrounding the sphenoid sinus and the clivus. Adjuvant radiotherapy was performed with a total dose of 59 Gy in 30 fractions. MRI 6 months post-radiosurgery revealed a persistence of the residual tumor surrounding the clivus with an extension to the floor of the sella turcica, the posterior part of the ethmoid sinus. Despite prior surgery and radiotherapy, the residual tumor persisted. Due to the anatomical complexity and prior radiation exposure, GKRS was selected by a multidisciplinary team to optimize local control. The treatment was performed in 2004 (PI of 50%, PD of 11 Gy, MD of 22 Gy). The post-operative course was uneventful and the patient was discharged home. The patient deceased in 2016, at 83 years old (12 years after GKRS); the cause of death was not related to the ossifying fibroma. (Figure 7).

### 3.5. Spindle Cell Oncocytoma (SCO)

**Figure 8:**
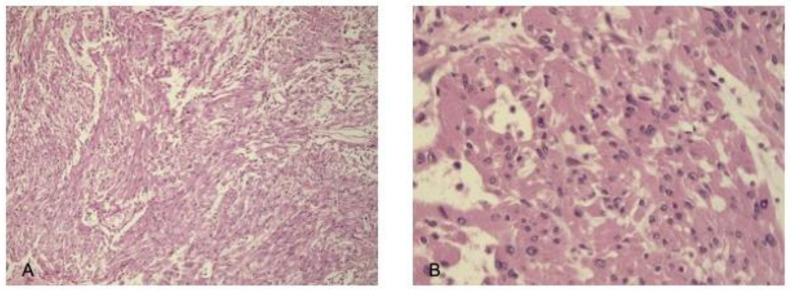
Microscopic appearance of spindle cell oncocytoma showing spindle cells ((**A**) hematoxylin and eosin stain; original magnification, ×100) with eosinophilic granular cytoplasm at higher magnification ((**B**) hematoxylin and eosin stain; original magnification, ×200).

Case 8. A 71-year-old woman presented with a short course of asthenia, occasional headache, lack of appetite, and unstable blood pressure, due to panhypopituitarism. The patient started hormonal replacement therapy, but soon after, she presented rapidly worsening visual field deficit. MRI (March 2012) showed a dilated sellar region due to presence of an expansive enhancing lesion. The patient underwent surgery through an endoscopic trans-nasal transsphenoidal approach. The histological findings are highlighted in Figure 8.

The post-operative course was uneventful and the patient was discharged home. Twelve months after surgery, the patient again showed visual field worsening due to the lesion’s relapse, so she underwent a second endoscopic surgical operation with a partial resection of the lesion. The patient recovered from the visual deficit after surgery and was finally addressed to GKRS for an adjuvant treatment in 2013 because a third operation was considered having too high risks (IP of 50%, Ds of 12 Gy, Dm of 24 Gy, integral dose of 38.8 mJ, 12 Gy volume of 2.67 cc). The clinical and radiological follow-up until 2019 (when the patient was lost in the post-COVID-19 era) revealed excellent tumor growth control, and the patient continued pharmacological replacement therapy for hypopituitarism without new neurological deficit.

### 3.6. Choroid Plexus Papilloma (CPP)

Case 9. A 56-year-old woman was admitted to our neurosurgery department due to persistent headache, the progressive deterioration of sight in the left eye, and disorders of equilibrium for 10 days. On neurological examination, the patient presented light dysarthria, gait ataxia, bilateral dysmetria, and a positive Romberg test. MRI of the brain revealed a regular-shaped lesion in the fourth ventricle with the initial dilation of the ventricular system. The patient underwent atlo-suboccipital craniectomy for the removal of the abovementioned lesion; the post-operative course was regular and she was discharged home 12 days after surgery. Histology revealed papillary structures with fibrovascular cores covered by one or more columnar epithelial cells; immunohistochemical analysis found the expression of cytokeratin but not glial fibrillary acid protein. Thus, the lesion was diagnosed as grade I CPP accordingly. Cerebral MRI after 8 years of follow-up showed the presence of a new lesion (diameters 11 × 12 mm) in the sellar–suprasellar region nearby the optic chiasm, lightly compressing the optic recess. The lesion appeared hypermetabolic upon CT-PET study. Considering the risks and the possible complications of a surgical approach, the patient underwent GKRS in 2011 (dose staging radiosurgery, covering the entire tumoral volume with a lower PD; first stage, therapy: PI of 50%, PD of 10 Gy, MD 20 of Gy, and PI of 50%; second stage: PD of 9 Gy and MD of 18 Gy after 1 month). One month after radiosurgery, the patient complained about persistent hiccup and dysphagia for solid and liquids, and MRI showed the sellar–suprasellar lesion (stable) and a new lesion located in the upper medulla (diameters 9.5 × 15 mm) with a cystic component that progressively increased in the following MRIs, so the patient underwent a second neurosurgical treatment through reopening of the previous suboccipital approach. Histology and immunohistochemical analysis indicated atypical grade II CPP (Ki-67 8–10%). The last MRI control confirmed the involution of the formation in the sellar region. The patient underwent visual field campimetry that showed bitemporal hemianopsia and reduced retina sensitivity in the lower external quadrant in both eyes. The patient died in 2019 because of liquoral seeding. Case data are summarized in Table 1.

## 4. Discussion

### 4.1. Pituitary Adenomas and Nonadenomatous Sellar Lesions

As is well known, pituitary adenomas are the most common tumors in the sellar region. Nevertheless, differential diagnosis might sometimes be difficult for other nonadenomatous lesions as well as craniopharyngiomas, Rathke’s cleft cysts, and spindle cell oncocytomas [2,7].

Rare lesions such as spindle cell oncocytoma, choroid plexus papillomas, and Rosai-Dorfman disease can furthermore complicate recognizing and to establishing adequate management [8,9].

Given this complexity, a multidisciplinary approach is essential for accurate diagnosis and optimal treatment.

Neuoradiological imaging, such as histopathology, is mandatory to distinguish these entities [10]; for example, cytokeratin profiles such as CK8 and CK20 can recognize craniopharyngiomas from Rathke’s cleft cysts [4].

### 4.2. Hypothalamic Hamartomas

Hypothalamic hamartomas are rare ectopic congenital lesions that are described as epileptogenic, particularly for those of the posterior hypothalamus and mammillary bodies [11,12], while anterior ones usually develop endocrinological impairments. Various classifications have been proposed for HHs but the most frequently used [13,14] are based on MR imaging findings considering the anatomical relationship between HH and the third ventricle [15]. Multiple neurosurgical approaches have been described for the management of epileptogenic hypothalamic hamartomas (HHs). Among these, Gamma Knife Radiosurgery (GKRS) has demonstrated efficacy as a minimally invasive option in selected pediatric patients with small type I or type III lesions. GKRS provides seizure control outcomes comparable to those of microsurgical resection, while offering a more favorable neurological risk profile. However, its primary limitation is the delayed onset of therapeutic effects, which necessitates long-term follow-up to adequately assess treatment response and clinical outcomes [16].

Management strategies include microsurgery, endoscopy, radiosurgery, and, more recently, focused ultrasounds, which demonstrate variable success rates in seizure control [14,17].

Radiosurgery, in particular Gamma Knife Radiosurgery, has been demonstrated to be safe and effective as a first-line therapy, effectively managing seizures while preserving endocrine function [12,13,18,19]. Cyber Knife Radiosurgery has also shown promise in managing severe epilepsy cases associated with HH [20], offering another non-invasive alternative.

### 4.3. Langerhans Cell Histiocytosis (LCH) and Other Histiocytosis

Langerhans cell histiocytosis is a rare idiopathic disease, characterized by the uncontrolled clonal proliferation of histiocytes in various tissues and organs, whose pathogenesis remains unclear. The incidence of LCH is 1–2 patients per million, and central nervous system invasion is exceedingly rare, especially in adults, since LCH usually involves pediatric patients, affecting the skull, column, femur, and pelvis with osteolytic lesions. Lesions’ locations within central nervous system (CNS) are crucial to establish the correct treatment [21].

LCH is another rare lesion that could affect the hypothalamic–pituitary axis and clinically result in endocrinopathies, including diabetes insipidus and hypopituitarism or anterior optic pathways [22].

Some cases present as isolated lesions in the optic chiasm or pituitary stalk, further complicating diagnosis [23]. Due to its variable presentation, early recognition and prompt intervention are crucial.

Conservative treatment may be considered for asymptomatic patients and lesions not involving critical areas [23]. High-dose corticosteroid therapy has been proposed for LCH-associated hypophysitis, but it is still controversial due to the side effects of the steroids [24]. Gamma Knife and Cyber Knife Radiosurgery have been proposed to control lesion growth [22]. Treatment modalities include salvage stereotactic radiotherapy for recurrent or persistent lesions, while systemic chemotherapy is required in multifocal disease presentations [25].

### 4.4. Rathke’s Cleft Cysts

Rathke’s cleft cysts (RCCs), also known as pars intermedia cysts, are benign lesions formed from remnants of the embryologic Rathke pouch. RCCs usually occupy the sellar space, located between the anterior and the posterior lobes of the pituitary gland [26]. These lesions are common incidental “asymptomatic” findings (present in 4–33% of autopsies), but they may account for 6–10% of symptomatic sellar and suprasellar lesions. The mean age of patients ranges from 40 to 50 and there is a tendency for female preponderance [3].

Symptomatic headaches, visual disturbances, hypopituitarism, amenorrhea, and galactorrhea are usually present at the moment of discovery. Hypophisitis has also been reported, induced by foreign body reaction to the contents of the cysts. Other symptoms are less frequently reported: hyponatriemia, aseptic meningitis, optic neuritis, diabetes insipidus, and cavernous sinus syndrome [26]. The critical location of RCCs makes their surgical treatment particularly challenging.

We proposed GKRS for RCCs with the aim of dramatically decreasing the risk of post-operative recurrences or progressions, thus avoiding both the need for repeat resections and the risk of further permanent complications. The rationale of including RCCs among the indications for GKRS arises from the fact that these lesions can be properly considered benign neoplastic forms [27]. In fact, some authors have suggested there is a close relationship between RCCs and craniopharyngioma since they share a common histological origin from remnants of squamous epithelium from Rathke’s cleft. In addition, the transformation from RCC to craniopharyngioma in the same patient has also been reported. Finally, it is well known that craniopharyngioma can be treated effectively with GKRS [5]. The authors showed that all the symptoms present before RS disappeared and neither endocrine dysfunctions nor tumor recurrences developed.

### 4.5. Other Forms

Ossifying fibroma is a fibro-osseus benign lesion firstly described in 1872 and most commonly seen in the head and neck region, but ethmoid sinus involvement is quite rare [28].

Ossifying fibroma (OF) is primarily a fibro-osseous lesion of the jawbones (mandible and maxilla) and the craniofacial skeleton. However, its occurrence in the sellar region is extremely rare, and only a few cases have been reported in the literature [29]. In the sellar region, ossifying fibroma may arise from embryonic remnants or mesenchymal tissue capable of bone formation. Its development in this area is not well understood, but it is likely associated with aberrant fibro-osseous activity within the cranial bones or paranasal sinuses [30].

The mean age of patients ranges from 20 to 30 years old with a slightly female prevalence (2:1). The etiology is unknown but some authors have suggested an origin from periodontal ligaments of teeth. Clinical presentation is variable depending on the tumor’s location, from asymptomatic, incidentally found lesions to nasal obstruction [29].

Spindle cell oncocytoma develops in the adenohypophyses and represents a surgical challenge due to its high recurrence rates and associated hemorrhagic risks [9,31].

Spindle cell oncocytoma is a recently described, benign, non-adenomatous tumor of adenohypophysis. SCO is a rare grade 1 WHO neoplasm which mostly affects adults aged 50–60 years with equal sex distribution. It primarily affects the sellar and suprasellar regions of the brain and typically arises from the anterior pituitary gland. It is composed of spindle-shaped cells with eosinophilic, oncocytic cytoplasm (hence the name “oncocytoma”). Its cells are organized in a fascicular or storiform pattern and show low mitotic activity and no significant atypia, which helps distinguish them from more aggressive tumors [31,32].

Only few previous cases of Stereotactic Radiosurgery for SCO have been reported, but GKR may be an effective alternative for recurrent cases [33].

However, further research is needed to determine long-term outcomes.

Similarly, Rosai–Dorfman disease, a benign histiocytic disorder, can mimic other sellar lesions, making accurate diagnosis essential.

Choroid plexus papillomas (CPPs) are a rare and slow-growing form of neuroectodermal tumor of the central nervous system, counting for less of 1% of all primary brain tumor, that originate from choroid plexus epithelial cells [34]. The most common location of these tumors is the trigon of the lateral ventricle (in infants and children) and the fourth ventricle (in adults). Nevertheless, in rare cases, they can develop in extra-ventricular sites such as the cerebellar–pontine angle, posterior fossa, brain stem, sacral canal, cerebral parenchyma, and suprasellar region [8,35,36]. In our series of unusual sellar region lesions, we report a case of sellar–suprasellar CPP radiologically mimicking a pituitary adenoma.

In addition to the rare sellar tumors treated in our series, other unusual lesions have also been successfully managed with radiosurgery, as reported in the literature. Vasquez et al. described the treatment of epidermoid tumors with Gamma Knife Radiosurgery (GKRS), highlighting its role in controlling growth and minimizing surgical risks associated with adherent cyst walls [37]. Similarly, Cohen-Inbar et al. presented a multicenter study on intracranial hemangiopericytoma, a typically aggressive neoplasm, where stereotactic radiosurgery offered promising local control rates with acceptable toxicity [38]. Albano et al. reported on primary sellar melanocytomas and other parasellar tumors, indicating that both primary radiosurgery and post-operative adjuvant radiotherapy, including stereotactic techniques, can be effective for tumors traditionally managed with challenging surgical resections [39].

These reports underline the evolving role of radiosurgical techniques as safe, effective, and minimally invasive options for a variety of rare lesions in the sellar and parasellar regions. Our findings, in line with these observations, suggest that GKRS should be considered a valuable treatment option not only for common sellar pathologies but also for a wider range of rare and histologically diverse tumors. Nevertheless, larger series and longer follow-ups are required to validate these preliminary favorable outcomes.

### 4.6. Radiotherapy as Salvage Therapy

Stereotactic radiation therapy, including Gamma Knife and Cyber Knife Radiosurgery, has emerged as a key approach for managing recurrent or refractory sellar lesions or in cases in which surgery is not feasible. These modalities offer high precision and spare critical structures, such as the pituitary stalk and anterior optic pathways [20,27].

Gamma Knife Radiosurgery provides highly focused radiation with minimal exposure to surrounding tissues, making it a preferred approach for localized tumors and lesions in this region especially.

Long-term studies emphasize, on the contrary, the importance of balancing therapeutic efficacy with potential side effects, in particularly radiation-induced endocrinopathies [19]. The meticulous follow-up of endocrine function is necessary in patients undergoing radiosurgery. Future advancements in radiosurgery, such as fractionation protocols, aim to further minimize complications, improving patient outcomes.

### 4.7. Emerging Insights and Research Needs

As seen before, variability in the clinical presentations and outcomes of rare sellar lesions highlights the need for multidisciplinary collaboration in treatment planning and decision-making [1,7].

Radiosurgery has also been proven to be safe and effective in cases of rare pituitary tumors and in cases of residuality/recurrence or patients not suitable for surgery.

## 5. Conclusions

The sellar and suprasellar regions are hosts to a diverse array of pathologies, each requiring a tailored diagnostic and therapeutic approach. Advances in imaging and radiosurgery, coupled with a deeper understanding of rare lesion biology, continue to expand the horizons for effective management. Integrating these modalities while maintaining a patient-centric focus remains the cornerstone of care.

## Figures and Tables

**Table 1:** Summary of patients’ data.

N	Histology	Treatment Parameters	Treatment Date—Last FU
1.	Hypothalamic Hamartoma	PI of 50%, PD of 11 Gy, MD of 22 Gy	2004–2020
2.	Hypothalamic Hamartoma	PI of 50%, PD of 13.5 Gy, MD of 27 Gy	2005–2010
3.	Hypothalamic Hamartoma	PI of 50%, PD of 13 Gy, MD of 26 Gy	2009–2019
4.	Langerhans Cell Histiocytosis (LCH)	PI of 50%, PD of 11 Gy, MD of 22 Gy	2004–2009
5.	Rathke’s Cleft Cyst	PI of 50%, PD of 12 Gy MD of 24 Gy	2007–2014
6.	Rathke’s Cleft Cyst	PI of 50%, PD of 10 Gy, MD of 20 Gy	2015–2023
7.	Ossifying Fibroma	PI of 50%, PD of 11 Gy, MD of 22 Gy	2004–2016
8.	Spindle Cell Oncocytoma	IP of 50%, Ds of 12 Gy, Dm of 24 Gy	2013–2019
9.	Ectopic Choroid Plexus Papilloma (CPP)	Dose Staging: PI of 50%, PD of 10 Gy, MD of 20 Gy, PI of 50%; PD of 9 Gy, MD of 18 Gy after 1 month	2011–2019

## Data Availability

Data are unavailable due to privacy or ethical restrictions.

## Abbreviations

GKRS: Gamma Knife Radiosurgery. HH: hypothalamic hamartoma. RCC: Rathke’s cleft cyst. LCH: Langerhans cell histiocytosis. SCO: spindle cell oncocytoma. CPP: choroid plexus papilloma. OF: ossifying fibroma. MRI: magnetic resonance imaging. PD: Prescription Dose. PI: Peripheral Isodose. MD: Maximum Dose. ECG: Electrocardiogram. CT-PET: Computed Tomography-Positron Emission Tomography. CNS: central nervous system. WHO: World Health Organization. H-E: hematoxylin and eosin. GK: Gamma Knife. FU: follow-up. MR: magnetic resonance. RS: radiosurgery. mJ: millijoule.
